# Several type 2 diabetes-associated variants in genes annotated to WNT signaling interact with dietary fiber in relation to incidence of type 2 diabetes

**DOI:** 10.1186/s12263-016-0524-4

**Published:** 2016-03-21

**Authors:** George Hindy, Inês G. Mollet, Gull Rukh, Ulrika Ericson, Marju Orho-Melander

**Affiliations:** Department of Clinical Sciences in Malmö, Lund University, Skåne University Hospital, Jan Waldenströms gata 35, SE-205 02 Malmö, Sweden

**Keywords:** Gene-diet interactions, TCF7L2, WNT signaling, Type 2 diabetes, Dietary fiber

## Abstract

**Background:**

TCF7L2 is a central transcription factor in the canonical wingless-type MMTV integration site (WNT) signaling pathway, and genetic variants in *TCF7L2* have been found to interact with dietary fiber intake on type 2 diabetes risk. Here, we investigate whether other type 2 diabetes genes could be involved in the WNT signaling pathway and whether variants in such genes might interact with dietary fiber on type 2 diabetes incidence.

**Results:**

We included 26,905 individuals without diabetes from the Malmö Diet and Cancer Study cohort. Diet data was collected at baseline using a food frequency questionnaire, a 7-day food record, and an interview. Altogether, 51 gene loci were analyzed for putative links to WNT signaling. Over a mean follow-up period of 14.7 years, 3132 incident cases of type 2 diabetes were recorded. Seven genes (nine single nucleotide polymorphisms (SNPs)) were annotated as involved in WNT signaling including *TCF7L2* (rs7903146 and rs12255372), *HHEX* (rs1111875), *HNF1A* (rs7957197), *NOTCH2* (rs10923931), *TLE4* (rs13292136), *ZBED3* (rs4457053), and *PPARG* (rs1801282 and rs13081389). SNPs in *TCF7L2*, *NOTCH2*, and *ZBED3* showed significant interactions with fiber intake on type 2 diabetes incidence (*P*_interaction_ = 0.034, 0.005, 0.017, and 0.002, respectively). The magnitude of the association between the *TCF7L2* risk allele and incident type 2 diabetes increased from the lowest to the highest quintiles of fiber intake. Higher fiber associated with lower type 2 diabetes risk only among risk allele carriers of the *NOTCH2* variant and homozygotes of the risk allele of the *ZBED3* variant.

**Conclusions:**

Our results suggest that several type 2 diabetes susceptibility SNPs in genes involved in WNT signaling may interact with dietary fiber intake on type 2 diabetes incidence.

**Electronic supplementary material:**

The online version of this article (doi:10.1186/s12263-016-0524-4) contains supplementary material, which is available to authorized users.

## Background

Type 2 diabetes is a multifactorial disease, and both genetic and environmental factors, and their complex interactions, are thought to contribute to the disease development. Recently, more than 80 type 2 diabetes associated genetic variants have been discovered mainly through genome-wide association studies (GWAS) [24, 28]. Of these, the transcription factor 7-like 2 gene (*TCF7L2*) variant rs7903146 shows the strongest association with type 2 diabetes [12].

TCF7L2 is a principal transcription factor in the canonical wingless-type MMTV integration site (WNT) signaling pathway [29]. This highlights the importance of the canonical WNT pathway in the pathogenesis of type 2 diabetes. Many studies have demonstrated an essential role for the WNT signaling pathway in pancreatic islet beta cell genesis and proliferation [33]. Glucagon-like peptide-1 (GLP-1)-mediated proliferation of rat beta cell line (INS-1) has been shown to occur through activation of the WNT pathway [19]. In addition, WNT signaling has been reported to mediate the expression of the pro-glucagon gene in intestinal L cells and thus the synthesis of GLP-1 [43]. Moreover, activation of the WNT pathway has been observed to prevent pre-adipocyte differentiation [32].

Several studies have reported a protective association between higher dietary fiber intake and type 2 diabetes [25, 39]. This inverse relationship seems to be likely driven by the cereal fiber component. In a meta-analysis of prospective studies, cereal fiber but not vegetable/fruit fiber intake has been shown to associate with lower risk of type 2 diabetes [34]. Short chain fatty acids (SCFA), the by-products of colonic fermentation of dietary fiber, have been reported to hyper-activate WNT signaling in a colorectal cell line [5]. Fiber intake could thus exert its protective effect at least partially by modifying WNT activity through SCFAs. SCFAs have previously been associated with increased insulin secretion [27], promotion of adipocyte differentiation [44], and induction of GLP-1 secretion via the free fatty acid receptor 2 (FFAR2) [36]. Biological interactions could conceivably occur between fiber intake through SFCAs and the WNT pathway on type 2 diabetes risk at the level of pancreatic beta cells, intestinal L cells, or adipocytes.

We have previously reported that the protective associated effect of higher fiber intake may be restricted to the non-risk CC genotype carriers of *TCF7L2* rs7903146 [14]. Similar results were also reported in the EPIC-Potsdam case-control study showing a protective association between higher whole grain intake and type 2 diabetes only among the non-risk allele carriers [9]. In line with these results, lower risk of type 2 diabetes by higher whole grain and cereal fiber intake was restricted to the non-risk allele carriers in a subgroup of men in the Stockholm Diabetes Prevention Program [42]. Further, another lifestyle intervention demonstrated a greater weight loss by higher fiber intake after a 9-month intervention among individuals carrying the non-risk CC genotype compared to risk allele carriers [13].

As association between *TCF7L2* and type 2 diabetes has highlighted an important role for WNT signaling in type 2 diabetes pathogenesis, we therefore used pathway analysis tools to evaluate whether other type 2 diabetes associated loci might be connected to this pathway. As several studies have indicated that dietary fiber or whole grain intake might modify the association between *TCF7L2* variants and type 2 diabetes or weight loss, we hypothesized that additional type 2 diabetes susceptibility genes in the WNT pathway may interact with fiber intake on type 2 diabetes incidence.

## Results

### Association between fiber intake and type 2 diabetes incidence and related quantitative traits

Association between fiber intake and type 2 diabetes incidence and related quantitative traits in the Malmö Diet and Cancer Study (MDCS) are shown in Table 1. A total of 3132 incident cases of type 2 diabetes were recorded after a mean follow-up time of 14.7 years. Higher fiber intake was observed to strongly associate (*P* < 0.001) with reduced baseline fasting plasma glucose (FPG), fasting plasma insulin (FPI), homeostasis model for assessment of insulin resistance (HOMA-IR), and glycated hemoglobin (HbA_1C_) (Table 1).

**Table 1:** Association between fiber intake and type 2 diabetes and related traits in MDCS

		Quintiles of fiber intake						
	Total	Q1	Q2	Q3	Q4	Q5	Βeta (SE) or HR (CI)	*P* value^a^
*N*	26,905	5381	5381	5381	5381	5381		
Sex (% males)	10,429 (39 %)	3039 (57 %)	2426 (45 %)	2062 (38 %)	1635 (30 %)	1267 (24 %)		
Age (years)	58.0 ± 7.6	57.4 ± 7.5	57.7 ± 7.7	58.2 ± 7.7	58.3 ± 7.7	58.3 ± 7.5		
Incident type 2 diabetes	3132 (10.6 %)	666 (11.3 %)	650 (11.0 %)	670 (11.4 %)	564 (9.56 %)	582 (10.9 %)	0.98 (0.96–1.01)	0.17
BMI (kg/m^2^)	25.6 ± 3.9	25.5 ± 3.9	25.6 ± 3.8	25.8 ± 3.9	25.7 ± 3.9	25.5 ± 4.0	0.02 (0.02)	0.22
FPG (mmol/L)^b^	5.64 ± 0.82	5.81 ± 1.04	5.68 ± 0.78	5.63 ± 0.79	5.58 ± 0.73	5.54 ± 0.72	−0.03 (0.008)	0.0001
FPI (pmol/L)^b^	46.5 ± 46.2	50.7 ± 43.5	46.2 ± 33.6	47.5 ± 50.5	45.1 ± 59.3	43.9 ± 38.9	−0.95 (0.48)	6 × 10^−07^
HOMA-IR^b^	1.60 ± 1.22	1.75 ± 1.32	1.64 ± 1.06	1.65 ± 1.62	1.53 ± 1.04	1.48 ± 1.00	−0.06 (0.01)	6 × 10^−09^
HbA_1C_ (%)^b^	4.81 ± 0.49	4.89 ± 0.58	4.82 ± 0.45	4.82 ± 0.53	4.80 ± 0.43	4.76 ± 0.47	−0.02 (0.005)	0.00002
HbA_1C_ (mmol/mol)^b^	39.7 ± 5.00	40.5 ± 5.88	39.8 ± 4.56	39.8 ± 5.36	39.5 ± 4.38	39.2 ± 4.75	−0.22 (0.05)	0.00002
Fiber (g/1000 Kcal)	9.03 ± 2.72	5.77 ± 0.81	7.47 ± 0.36	8.69 ± 0.36	10.09 ± 0.48	13.13 ± 2.19		

Data are means ± SD unless otherwise stated. Number of individuals included in MDCS cohort, *n* = 26,905

*HR* hazard ratio, *BMI* body mass index, *FPG* fasting plasma glucose, *FPI* fasting plasma insulin, *HOMA-IR* homeostatic model assessment for insulin resistance, *HbA*_*1C*_ glycated hemoglobin

^a^Adjusted for age, sex, BMI, total energy intake, dietary assessment method, season, leisure-time physical activity, smoking, alcohol intake, and education where appropriate

^b^Data available only for the MDC-CC, *n* = 5499

### Annotation of type 2 diabetes associated genes to the WNT pathway

A total of nine type 2 diabetes associated genes were annotated to the WNT signaling pathway, and of these, seven genes (nine single nucleotide polymorphisms (SNPs)) were annotated and selected for further analyses. We included SNPs mapping to genes with roles upstream of TCF7L2 in the WNT signaling pathway. Functional positioning of these genes within the WNT pathway is detailed in the Additional file 1: Figure S1. These included *TCF7L2* (rs7903146 and rs12255372), *HHEX* (rs1111875), *HNF1A* (rs7957197), *NOTCH2* (rs10923931), *TLE4* (rs13292136), *ZBED3* (rs4457053), and *PPARG* (rs1801282 and rs13081389).

### Association between WNT-annotated gene variants and type 2 diabetes and related traits

Both *TCF7L2* variants (rs7903146 and rs12255372), *HHEX* rs1111875, and *HNF1A* rs7957197 were associated with increased risk of type 2 diabetes (Table 2). In addition, the *TCF7L2* variants and the *HNF1A* variant associated with elevated baseline FPG. The *HHEX* variant associated with lower baseline FPI, and the *TCF7L2* variants and the *ZBED3* variant associated with elevated baseline HbA_1C_ (Table 2).

**Table 2:** Association between SNPs in WNT-associated genes and type 2 diabetes and related traits in MDCS

	Incident type 2 diabetes^a^		FPG (mmol/L)^b^		FPI (pmol/L)^b^		HbA_1C_ (%)^b^	
	HR (95 % CI)	*P* value	Beta (SE)	*P* value	Beta (SE)	*P* value	Beta (SE)	*P* value
*TCF7L2* rs7903146	1.33 (1.25–1.40)	1 × 10^−23^	0.04 (0.02)	0.03	0.16 (1.05)	0.82	0.02 (0.01)	0.04
*TCF7L2* rs12255372	1.24 (1.17–1.31)	9 × 10^−14^	0.03 (0.02)	0.15	−0.73 (1.07)	0.79	0.02 (0.01)	0.07
*HHEX* rs1111875	1.06 (1.01–1.12)	0.03	−0.02 (0.02)	0.20	−3.07 (0.99)	0.016	0.01 (0.01)	0.31
*HNF1A* rs7957197	1.12 (1.05–1.20)	0.001	0.04 (0.02)	0.04	1.11 (1.23)	0.54	0.01 (0.01)	0.30
*NOTCH2* rs10923931	0.99 (0.91–1.09)	0.90	0.04 (0.03)	0.19	−0.14 (1.73)	0.69	0.02 (0.02)	0.35
*TLE4* rs13292136	1.03 (0.94–1.12)	0.60	−7 × 10^−05^ (0.03)	1.00	1.71 (1.72)	0.40	0.02 (0.02)	0.30
*ZBED3* rs4457053	1.01 (0.96–1.08)	0.64	0.01 (0.02)	0.44	−1.41 (1.14)	0.22	0.02 (0.01)	0.04
*PPARG* rs1801282	1.08 (1.00–1.16)	0.051	0.03 (0.02)	0.24	1.16 (1.38)	0.42	−0.002 (0.01)	0.88
*PPARG* rs13081389	1.00 (0.91–1.11)	0.97	0.03 (0.03)	0.35	1.67 (1.79)	0.33	0.01 (0.02)	0.68

*HR* hazard ratio, *FPG* fasting plasma glucose, *FPI* fasting plasma insulin, *HbA*_*1C*_ glycated hemoglobin

^a^Adjusted for age and sex, *N* = 26,905

^b^Adjusted for age and sex; data available only for the MDC-CC, *N* = 5499

### Interaction between WNT-annotated gene variants and fiber intake on incidence of type 2 diabetes

The *TCF7L2* rs7903146 variant interacted with quintiles of fiber intake on type 2 diabetes incidence (*P*_interaction_ = 0.034). Additionally, another *TCF7L2* variant, rs12255372, showed a somewhat stronger interaction with fiber intake on type 2 diabetes incidence (*P*_interaction_ = 0.005) (Table 3). The magnitude of the associated effect of the *TCF7L2* risk allele on type 2 diabetes incidence was higher among individuals in the higher fiber intake quintiles as compared to individuals in the lower quintiles. Moreover, individuals in the lowest fiber quintile did not show any increased risk of type 2 diabetes by the risk (T) allele of rs12255372 (Table 4). Higher fiber intake showed a tendency for association with lower risk of type 2 diabetes among non-risk GG genotype carriers of rs12255372 (hazard ratio (HR) 0.96; 95 % confidence interval (CI) 0.92–1.00; *P* = 0.06) but not among GT and TT genotype carriers.

**Table 3:** Interaction between variants in WNT-associated genes and quintiles of fiber intake on type 2 diabetes incidence

			Genotype			HR^b, c, d, e^	95 % CI	*P* ^b, c, d, e^
	Alleles	Risk allele	XX	XR^a^	RR			
*TCF7L2* rs7903146	C/T	T	13,475	9414	1720	1.20^c^	1.05–1.36	0.006
Fiber						0.96^d^	0.93–0.998	0.037
*TCF7L2* rs7903146 × Fiber						1.04^e^	1.003–1.09	0.034
*TCF7L2* rs12255372	G/T	T	13,517	9689	1785	1.06	0.93–1.21	0.36
Fiber						0.95	0.91–0.98	0.003
*TCF7L2* rs12255372 × Fiber						1.06	1.02–1.10	0.005
*HHEX* rs1111875	A/G	G	4329	12,518	8816	0.99	0.88–1.11	0.83
Fiber						0.95	0.90–1.00	0.05
*HHEX* rs1111875 × Fiber						1.03	0.99–1.07	0.14
*HNF1A* rs7957197	A/T	T	1067	8549	16,401	1.15	0.99–1.34	0.06
Fiber						1.00	0.92–1.08	0.92
*HNF1A* rs7957197 × Fiber						0.99	0.95–1.04	0.75
*NOTCH2* rs10923931	G/T	T	20,612	4372	228	1.24	1.02–1.51	0.035
Fiber						1.00	0.97–1.03	0.97
*NOTCH2* rs10923931 × Fiber						0.93	0.87–0.99	0.017
*TLE4* rs13292136	C/T	C	186	3928	21,962	0.99	0.81–1.23	0.96
Fiber						0.97	0.86–1.10	0.66
*TLE4* rs13292136 × Fiber						1.01	0.94–1.07	0.88
*ZBED3* rs4457053	A/G	G	13,455	9362	1642	1.22	1.06–1.39	0.004
Fiber						1.02	0.98–1.05	0.38
*ZBED3* rs4457053 × Fiber						0.94	0.90–0.98	0.002
*PPARG* rs1801282	G/C	C	548	6263	19,038	1.18	0.99–1.40	0.06
Fiber						1.01	0.92–1.12	0.77
*PPARG* rs1801282 × Fiber						0.98	0.93–1.04	0.52
*PPARG* rs13081389	A/G	A	165	3256	21,423	0.97	0.77–1.21	0.77
Fiber						0.93	0.81–1.07	0.30
*PPARG* rs13081389 × Fiber						1.03	0.96–1.10	0.46

*HR* hazard ratio

^a^X designates the non-risk allele and R designates the risk allele

^b^Adjusted for age, sex, BMI, total energy intake, dietary assessment method, season, leisure-time physical activity, smoking, alcohol intake, and education

^c^Main effect per risk allele from the multivariable model including the interaction term

^d^Main effect per quintile of fiber intake from the multivariable model including the interaction term

^e^Interaction effect

**Table 4:** HR of incident type 2 diabetes by genotype and quintiles of fiber intake

		Genotype					
		XX	XR^a^	RR	HR per R allele	*P*_trend_^b^	*P*_interaction_^c^
*TCF7L2* rs7903146							
	Q1	1.16 (0.98–1.39)	1.58 (1.32–1.89)	1.56 (1.14–2.15)	1.24 (1.09–1.40)	0.001	0.034
	Q2	1.13 (0.95–1.35)	1.52 (1.27–1.82)	1.92 (1.43–2.60)	1.26 (1.11–1.42)	0.0002	
	Q3	1.15 (0.97–1.37)	1.62 (1.36–1.93)	2.10 (1.60–2.77)	1.37 (1.22–1.55)	1 × 10^−7^	
	Q4	1.02 (0.85–1.22)	1.35 (1.12–1.62)	1.93 (1.48–2.58)	1.31 (1.15–1.49)	5 × 10^−5^	
	Q5	1 (ref)	1.70 (1.42–2.03)	2.00 (1.49–2.70)	1.47 (1.30–1.66)	2 × 10^−9^	
	HR per Q	0.97 (0.93–1.01)	0.99 (0.95–1.03)	1.05 (0.95–1.15)			
	*P*_trend_^c^	0.19	0.64	0.37			
*TCF7L2* rs12255372							
	Q1	1.22 (1.03–1.45)	1.44 (1.20–1.73)	1.27 (0.90–1.78)	1.08 (0.95–1.22)	0.26	0.005
	Q2	1.16 (0.98–1.38)	1.32 (1.10–1.58)	1.74 (1.31–2.32)	1.16 (1.02–1.31)	0.02	
	Q3	1.07 (0.90–1.28)	1.53 (1.29–1.82)	1.89 (1.44–2.47)	1.36 (1.21–1.53)	3 × 10^−7^	
	Q4	1.02 (0.86–1.22)	1.27 (1.05–1.52)	1.68 (1.25–2.26)	1.23 (1.08–1.40)	0.002	
	Q5	1 (ref)	1.41 (1.18–1.68)	1.99 (1.48–2.67)	1.40 (1.23–1.59)	4 × 10^−7^	
	HR per Q	0.96 (0.92–1.00)	0.98 (0.94–1.02)	1.05 (0.96–1.16)			
	*P*_trend_	0.060	0.37	0.28			
*NOTCH2* rs10923931							
	Q1	1.00 (0.87–1.14)	1.14 (0.92–1.42)	1.66 (0.86–3.23)	1.12 (0.93–1.34)	0.24	0.017
	Q2	1.00 (0.88–1.14)	1.04 (0.85–1.28)	0.76 (0.28–2.04)	1.06 (0.88–1.28)	0.51	
	Q3	1.03 (0.91–1.18)	1.10 (0.90–1.35)	1.05 (0.47–2.34)	1.08 (0.90–1.30)	0.41	
	Q4	0.97 (0.85–1.11)	0.70 (0.55–0.90)	0.75 (0.28–2.00)	0.75 (0.60–0.95)	0.015	
	Q5	1 (ref)	1.00 (0.80–1.25)	0.25 (0.04–1.80)	0.93 (0.75–1.15)	0.50	
	HR per Q	1.00 (0.97–1.03)	0.93 (0.87–0.996)	0.63 (0.44–0.90)			
	*P*_trend_	0.89	0.038	0.010			
*ZBED3* rs4457053							
	Q1	0.96 (0.81–1.13)	1.03 (0.86–1.23)	1.51 (1.14–2.01)	1.16 (1.02–1.31)	0.06	0.002
	Q2	0.96 (0.81–1.12)	1.00 (0.84–1.20)	1.04 (0.74–1.46)	1.03 (0.90–1.17)	0.36	
	Q3	1.04 (0.89–1.21)	1.04 (0.88–1.24)	0.85 (0.59–1.22)	0.99 (0.87–1.12)	0.97	
	Q4	0.93 (0.79–1.09)	0.93 (0.78–1.12)	0.67 (0.46–0.99)	0.95 (0.83–1.10)	0.55	
	Q5	1 (ref)	0.98 (0.82–1.17)	0.72 (0.50–1.04)	0.94 (0.81–1.07)	0.25	
	HR per Q	1.01 (0.97–1.05)	0.98 (0.94–1.03)	0.85 (0.76–0.95)			
	*P*_trend_	0.69	0.40	0.003			

*HR* hazard ratio, *Q* quintile

^a^X designates the non-risk allele and R designates the risk allele

^b^Adjusted for age and sex

^c^Adjusted for age, sex, BMI, total energy intake, dietary assessment method, season, leisure-time physical activity, smoking, alcohol intake, and education

Of the WNT-annotated SNPs (in genes upstream of TCF7L2), we found, in addition to *TCF7L2*, the SNPs in *NOTCH2* and *ZBED3* loci (Table 4) to significantly interact with fiber intake on type 2 diabetes incidence. The *NOTCH2* locus was observed to interact with fiber intake on type 2 diabetes incidence (*P*_interaction_ = 0.017). Higher fiber intake was observed to associate with lower risk of type 2 diabetes only among carriers of the risk (T) allele; GT (HR 0.93; CI 0.87–0.996; *P* = 0.038); and TT (HR 0.63; CI 0.44–0.90; *P* = 0.01). The *NOTCH2* risk allele did not associate with type 2 diabetes incidence in the whole population (HR 0.99; CI 0.91–1.09; *P* = 0.90); however, it was associated significantly with decreased risk of type 2 diabetes in the fourth fiber intake quintile (HR 0.75; CI 0.60–0.95; *P* = 0.015) (Table 4). Another interaction was observed between quintiles of fiber intake and *ZBED3* rs4457053 on type 2 diabetes incidence (*P*_interaction_ = 0.002). Higher fiber intake was observed to associate with lower risk of type 2 diabetes only among homozygotes for the risk (G) allele (HR 0.85; CI 0.76–0.95; *P* = 0.003) (Table 4).

## Discussion

In this prospective study, we observe interactions between dietary fiber intake and variants in three genes annotated to the canonical WNT signaling pathway influencing the risk of type 2 diabetes in a large population-based cohort over a 15-year follow-up period. This indicates a putative role of the WNT pathway in the pathogenesis of type 2 diabetes and a potential modulating role of dietary fiber through this pathway.

Annotation of the 51 gene loci corresponding to 58 SNPs associated with type 2 diabetes resulted in identification of seven genes having known connections with WNT signaling. We could replicate our previous finding of interaction between *TCF7L2* variants and fiber intake in relation to the risk of developing type 2 diabetes over time [14]. In addition, novel interactions between dietary fiber intake and two type 2 diabetes-associated variants in the *NOTCH2* and *ZBED3* loci were observed. Higher fiber intake was observed to associate with lower incidence of type 2 diabetes only among risk allele carriers of the *NOTCH2* variant and homozygotes for the risk allele of the *ZBED3* variant.

The WNT pathway may play a central role in the pathogenesis of type 2 diabetes. Pancreatic beta cell genesis and GLP-1-mediated proliferation have been shown to be mediated through activation of the WNT pathway [19, 33]. In addition to effects on beta cells, the WNT pathway mediates pro-glucagon gene expression in intestinal L cells and thus the synthesis of GLP-1 [43]. Activation of the WNT pathway has been shown to keep pre-adipocytes in their undifferentiated state and could eventually result in ectopic fat accumulation and insulin resistance [32]. Recently, a liver-specific knock out of *tcf7l2* in mice was linked to reduced hepatic glucose production [4]. WNT transcriptional activity has been reported to be hyper-activated by butyrate which is one of the SCFAs produced by microfloral fermentation of dietary fiber leading to apoptosis of colorectal cancer cells [5, 17]. Fiber intake is mainly linked to elevated luminal concentrations of SCFAs in the intestines. In addition, it has been linked to elevated SCFAs in plasma after 1 year of high-fiber intervention [10]. Fiber intake could thus at least partially exert its systemic anti-diabetogenic effects through modulation of the WNT signaling pathway by SCFAs.

In line with our previous report, we observed an interaction between *TCF7L2* rs7903146 and fiber intake on type 2 diabetes incidence. However, our present study included 3132 type 2 diabetes cases instead of 1649 cases in our previous study [14]. Another *TCF7L2* SNP (rs12255372) showed a somewhat stronger interaction with fiber. However, the two SNPs are in linkage disequilibrium (*r*^2^ = 0.70 and *D*’ = 0.91) and are thus very likely reflecting the same interaction. In this study, we observe that the direction of the interaction effects was in opposite direction with variants in *NOTCH2* and *ZBED3* as compared to the *TCF7L2* variants. Reports from colon cancer research show that the WNT pathway should not be viewed as having an additive effect but rather as a continuum since both high and low WNT activity has been shown to promote colorectal cancer cell apoptosis [5, 17]. The dual role played by TCF7L2 adds to this complexity where it can act as a transcriptional activator in the presence of beta catenin or as a repressor in the absence of beta catenin [3, 31]. This highlights the complexity by which WNT signaling may operate in the pathogenesis of type 2 diabetes and could explain the directional discrepancies observed in the interactions with fiber. Both *NOTCH2* and *ZBED3* variants showed no association with type 2 diabetes risk in MDCS with HRs close to null. Although this could be partly because of power limitations due to the smaller sample size compared to large-scale GWASs, the results of our interaction analyses may indicate that environmental factors such as higher fiber intake may in fact attenuate and possibly reverse the risk associated with these genetic variants.

The *TCF7L2* rs7903146 shows the strongest association with type 2 diabetes, as compared to more than 65 other loci identified to date among Europeans, with each risk allele of rs7903146 associating with around 40 % elevated risk of type 2 diabetes [28]. Most of the studies so far have reported beta cell dysfunction and an attenuated incretin effect associated with the risk allele [21,37]. A 1-year high-fiber intervention was linked to elevated plasma SCFAs and GLP-1 in hyperinsulinemic subjects [10]. Furthermore, SCFAs have been shown to induce GLP-1 secretion via the FFAR2 in mice [36]. Therefore, the carriers of the *TCF7L2* risk allele may suffer from beta cell incretin resistance and thus could be less prone to the beneficial associated effects of higher fiber intake through increased GLP-1 secretion. SCFAs, and particularly butyrate, are potent histone deacetylase inhibitors (HDACi), and this is particularly important as the *TCF7L2* variant has been linked to an islet-specific open chromatin state which could be exacerbated by higher fiber intake [11]. ZBED3 has been recently identified as an axin-binding protein that modulates the WNT signaling pathway [8]. Binding of the ZBED3 to axin prevents the formation of the destruction complex that normally phosphorylates beta catenin and targets it for proteasomal degradation. *ZBED3* risk allele has been shown to be associated with increased ZBED3 expression in adipose tissue, and this could translate to elevated WNT activity [38]. The *NOTCH2* gene encodes a transmembrane protein which physically associates with active beta catenin and decreases its levels leading to lower WNT activity in both stem and colon cancer cells [16]. However, whether the *NOTCH2* variant associates with NOTCH2 expression is not yet known.

The high relative validity of our dietary assessment method, the combination of a diet diary with a questionnaire, the large sample size, and the prospective design with a long follow-up period are the major strengths of our study. In addition, the detailed and extensive ascertainment of type 2 diabetes cases from more than six registers is another strength that decreases error and improves the power of our study. However, our study has several limitations that need to be discussed. First of all, the T2D gene loci used for annotation were obtained from the GWASs which in most cases are based on proximity to the SNPs and are not necessarily the causal genes. Further, although our study population is large, it still has a limited power for detecting interactions which could result in many false negative outcomes. Therefore, replication of our results in other studies with reasonable power and good quality of diet data is important. We did not correct for multiple testing as our study is hypothesis driven, and we believe that the selected WNT-associated genes are biologically dependent and the interactions with the *NOTCH2* and *ZBED3* variants may reflect essentially the interactions with *TCF7L2*. Finally, our diet data was collected at baseline and may fail to detect the effect of the changes in dietary habits over the follow-up period.

## Conclusions

Our results indicate that several type 2 diabetes susceptibility SNPs in genes involved in WNT signaling may interact with dietary fiber intake on type 2 diabetes incidence. The putative mechanisms by which fiber intake could modify WNT signaling through these variants in type 2 diabetes pathogenesis include effects on beta cell survival, adipogenesis and adipocyte proliferation, and/or GLP-1 production in intestinal L cells. In addition, functional studies are needed to better understand the putative role played by dietary fiber in type 2 diabetes through the WNT signaling pathway.

## Methods

### Study population

The MDCS is a population-based prospective cohort in the city of Malmö in Sweden. The source population (74,138 individuals) was defined in 1991 to include all individuals living in Malmö and born between the years 1926 and 1945. In 1995, this definition was modified to include all men born between 1923 and 1945 and women born between 1923 and 1950 [2, 22]. Study participants were recruited through public advertisements or personal letters. Individuals with mental disability and limited Swedish language skills were excluded. Study participants visited the screening center twice during the baseline examination period between March 1991 and October 1996. During the first visit, participants received instructions on recording their meals in the menu book and on filling in the diet questionnaire and the extensive questionnaire covering socioeconomic and lifestyle factors. Approximately 10 days later, subjects returned for a diet history interview. By the end of the baseline examination period, complete dietary, anthropometric and lifestyle data, was collected for 28,098 participants. Details of the recruitment procedures are described elsewhere [23].

After exclusion of prevalent type 2 diabetes patients, identified as individuals with a self-reported diabetes diagnosis or on a self-reported anti-diabetic regimen, we were left with 26,905 individuals for our prospective analyses. From the MDCS, 6103 individuals were randomly selected to participate in a cardiovascular sub-cohort (Malmö Diet and Cancer Study Cardiovascular Cohort (MDC-CC)). Additional measurements were obtained for these individuals, including analysis of FPG, FPI, and HbA_1C_ levels. After excluding prevalent cases of diabetes and individuals with no diet data, this sub-cohort consisted of 5499 individuals.

The MDCS was approved by the Ethical Committee at Lund University (LU 51-90). All participants provided written informed consent.

### Incident type 2 diabetes

Incident cases of type 2 diabetes were identified using the nationwide Swedish National Diabetes Register [7], the regional Diabetes 2000 register of the Scania region [18], or the Swedish National Patient Register, which is a principal source of data for numerous research projects and which covers more than 99 % of all somatic and psychiatric hospital discharges and Swedish hospital-based outpatient care [20]. Individuals could also be classified as diabetes cases if their cause of death was registered as diabetes in the Swedish Cause-of-Death Register, which comprises all deaths in Swedish residents occurring in Sweden or abroad [15], if they had been prescribed anti-diabetic medication according to the Swedish Prescribed Drug Register [40], or if they had at least two HbA_1C_ recordings ≥6.0 % using the Swedish Mono-S standardization system (corresponding to 7.0 % according to the US National Glycohemoglobin Standardization Program (NGSP) and 52 mmol/mol using International Federation of Clinical Chemistry and Laboratory Medicine units) in the Malmö HbA_1C_ register, which analyzed and cataloged all HbA_1C_ samples at the Department of Clinical Chemistry taken in institutional and non-institutional care in the greater Malmö area from 1988 onwards.

In addition, diabetes cases at the baseline examination of MDC-CC were identified based on self-reports of a physician diagnosis or the use of diabetes medication in a baseline questionnaire, or fasting whole blood glucose of ≥6.1 mmol/L (corresponding to FPG concentration of ≥7.0 mmol/L). Furthermore, a diabetes diagnosis could be determined at the MDC-CC reinvestigation between the years 2007 and 2012 based on self-reports of a physician diagnosis or the use of diabetes medication based on a questionnaire or FPG of ≥7.0 mmol/L or a 120-min value post-OGTT plasma glucose of >11.0 mmol/L. A total of 3132 incident cases of type 2 diabetes were identified during the mean follow-up period of 14.7 years until December 2010.

### Dietary assessment

A modified dietary history method specifically designed for the MDCS was used consisting of a 7-day menu book, a dietary 168-item questionnaire, and an interview. In the menu book, participants were asked to record lunch and dinner meals, cold beverages including alcohol, medicinal drugs, natural remedies, and nutrient supplements. The 168-item questionnaire was used to assess meal patterns, consumption frequencies, and portion sizes of regularly consumed foods. A 48-page booklet was used to help participants at home estimate the portion sizes for the questionnaire. During the interview, a more extensive book with photographs was used to estimate portion sizes and dishes in the menu book. Participants were also asked about their meal pattern, cooking methods, and food choices.

The average daily intake of foods was calculated using data from the menu book and diet questionnaire. The MDC Food and Nutrient Database, which was derived from PC KOST2-93 of the Swedish National Food Administration, was used to convert the average daily food intake to energy and nutrient intakes [6, 41].

A slight alteration of the coding routines for dietary data was introduced in September 1994 [41]. A method variable, classifying data collected before and after September 1994, along with a four-category season variable (i.e., winter, spring, summer, and autumn) was created and used as covariate to adjust for variation in data collection over time.

Dietary variables used in our analysis included total energy intake and dietary fiber. Fiber intake was adjusted for total energy intake by expressing it as grams per 1000 kcal. The relative validity of the dietary assessment method in MDCS has previously been evaluated in a sample of 50- to 69-year-old Malmö residents, 105 women, and 101 men. The reference method used was 18 days’ weighed food records (3 days every second month) collected over 1 year. Energy-adjusted Pearson correlation coefficients for fiber intake was 0.69 for women and 0.74 for men [30].

### Other variables used as potential confounders

An extensive lifestyle questionnaire adapted from the Minnesota Leisure Time Physical Activity Questionnaire was used to assess leisure-time physical activity. Participants had to estimate the number of minutes per week for each season they spent performing each of 17 different physical activities. The duration was multiplied by an intensity factor to create a physical activity score that was divided into tertiles. Alcohol intake was classified into four categories based on grams of alcohol consumed per day: zero-consumers, low (<15 g/day in women or <20 g/day in men), medium (15–30 g/day in women or 20–40 g/day in men), and high consumers (>30 g/day in women or >40 g/day in men). Participants were classified as current smokers, ex-smokers, and never-smokers. The education variable was created by classifying participants according to their highest educational level (≤8 years, 9–10 years, and 11–13 years at school and university degree).

### Genotyping

A total of 58 previously reported type 2 diabetes-associated single nucleotide polymorphisms were selected for genotyping in the MDCS (Additional file 1: Table S1) [24, 28]. Genotyping was performed by MassARRAY iPLEX (Sequenom, San Diego, CA, USA) or Taqman 5′ nuclease (Applied Biosystems, Foster City, CA, USA) assays according to the manufacturers’ instructions. The concordance rate was >99 % in 5500 samples which were genotyped using Human Omni Express Exome Bead Chip Kit (Illumina, San Diego, CA, USA). Genotyping success rate ranged between 96 and 99 %. All genotypes were in the Hardy-Weinberg equilibrium (*P* > 0.0009).

### Gene annotation

A list of 51 unique gene loci, corresponding to 58 SNPs (MDCS) associated with type 2 diabetes, were analyzed for links to WNT signaling (Additional file 1: Table S2) using a combination of annotation from the Gene Ontology [1], the Kyoto Encyclopedia of Genes and Genomes (http://www.kegg.jp/kegg/, accessed on the 15th of March 2013), Biocarta pathways (http://cgap.nci.nih.gov/Pathways/BioCarta_Pathways, accessed on the 15th of March 2013), Panther pathways [26, 35], and Literature integrated within the DAVID Annotation Bioinformatics Resource (http://david.abcc.ncifcrf.gov, accessed on the 15th of March 2013).

### Statistical analysis

Assuming an additive model, Cox proportional hazards regression was used to calculate the HR of incident type 2 diabetes associated with each risk allele in the MDCS adjusting for age and sex. After stratification to quintiles of relative intakes of fiber (g/1000 kcal), interactions between the SNP genotypes and quintiles of fiber intakes on type 2 diabetes incidence were analyzed by introducing a multiplicative factor of the genotype and dietary quintiles as continuous variables in addition to these variables to the equation. Interactions were analyzed adjusting for age, sex, BMI, total energy intake, dietary assessment method, season leisure-time physical activity, alcohol intake, smoking habits, and level of education. In the MDC-CC sub-cohort, we performed cross-sectional analysis using linear regression to calculate the effect sizes of each WNT-annotated risk allele on baseline FPG, FPI, HOMA-IR, and HbA_1C_ adjusting for age and sex.

We used IBM SPSS Statistics, version 19 (IBM Corporation, Armonk, NY, USA), for the analyses. Two-sided *P* values of <0.05 were considered significant.

### Compliance with ethics guidelines

All procedures followed were in accordance with the ethical standards of the responsible committee on human experimentation (Ethical Committee at Lund University LU 51-90) and with the Helsinki Declaration of 1975, as revised in 2000. Informed consent was obtained from all participants for being included in the study.

### Availability of supporting data

Two supplementary tables and a supplementary figure are included in the online resource.

## Additional file

Additional file 1: Table S1–S2 and Figure S1. Table S1. Type 2 diabetes associated loci. Table S2. Canonical WNT signaling genes. Figure S1. Functional positioning of ZBED3, TLE4, TCF7L2, HNF1A, HHEX, PPARG and NOTCH2 within the WNT Canonical pathway. (PDF 1268 kb)

## Abbreviations

CI: confidence interval
FFAR2: free fatty acid receptor 2
FPG: fasting plasma glucose
FPI: fasting plasma insulin
GLP-1: glucagon-like peptide-1
GWAS: genome-wide association study
HbA_1C_: glycated hemoglobin
HOMA-IR: homeostasis model for assessment of insulin resistance
HR: hazard ratio
MDC-CC: Malmö Diet and Cancer Study Cardiovascular Cohort
MDCS: Malmö Diet and Cancer Study
SCFA: short chain fatty acid
SNP: single nucleotide polymorphism
TCF7L2: transcription factor 7-like 2 gene
WNT: wingless-type MMTV integration site
