# Supplementary material for: Several type 2 diabetes-associated variants in genes annotated to WNT signaling interact with dietary fiber in relation to incidence of type 2 diabetes
Source: Genes Nutr. 2016 Mar 21;11:6. doi: 10.1186/s12263-016-0524-4 (PMC4968454; doi:10.1186/s12263-016-0524-4)
Supplement: Additional file 1: Table S1–S2 and Figure S1. — Table S1. Type 2 diabetes associated loci. Table S2. Canonical WNT signaling genes. Figure S1. Functional positioning of ZBED3, TLE4, TCF7L2, HNF1A, HHEX, PPARG and NOTCH2 within the WNT Canonical pathway. (PDF 1268 kb) [file 12263_2016_524_MOESM1_ESM.pdf]

Supplementary information

Genes and Nutrition

Several type 2 diabetes associated variants in genes annotated to WNT signaling interact with dietary fiber in relation to incidence of type 2 diabetes

George Hindy, Inês G. Mollet, Gull Rukh, Ulrika Ericson, Marju Orho-Melander

Department of Clinical Sciences in Malmö, Lund University, Skåne University Hospital, Jan Waldenströms gata 35, SE-205 02, Malmö, Sweden

Corresponding author:

George Hindy
Phone: +46 40 39 12 48
Fax: +46 40 39 12 22
E-mail: george.hindy@med.lu.se

Table S1. Type 2 diabetes associated loci

rsID	Locus	Major /Minor	MAF	Risk Allele	References
rs2943641	IRS1	C/T	0.37	C	(Rung et al. 2009)
rs1801282	PPARG	C/G	0.14	C	(Saxena et al. 2007)
rs13081389	PPARG	A/G	0.12	A	(Voight et al. 2010)
rs7903146	TCF7L2	C/T	0.26	T	(Grant et al. 2006)
rs12255372	TCF7L2	G/T	0.2	T	(Grant et al. 2006)
rs9939609	FTO	T/A	0.45	A	(Frayling et al. 2007)
rs243021	BCL11A	C/T	0.46	T	(Voight et al. 2010)
rs4457053	ZBED3	A/G	0.26	G	(Voight et al. 2010)
rs11634397	ZFAND6	G/A	0.4	G	(Voight et al. 2010)
rs13292136	TLE4	C/T	0.07	C	(Voight et al. 2010)
rs1531343	HMGA2	G/C	0.9	C	(Voight et al. 2010)
rs1552224	CENTD2	T/G	0.12	T	(Voight et al. 2010)
rs2191349	DGKB	T/G	0.48	T	(Dupuis et al. 2010)
rs340874	PROX1	G/A	0.48	G	(Dupuis et al. 2010)
rs780094	GCKR	G/A	0.48	G	(Dupuis et al. 2010; Saxena et al. 2007)
rs7957197	HNF1A	T/A	0.15	T	(Voight et al. 2010)
rs8042680	PRC1	C/A	0.22	A	(Voight et al. 2010)
rs896854	TP53INP1	G/A	0.48	A	(Voight et al. 2010)
rs972283	KLF14	G/A	0.45	G	(Voight et al. 2010)
rs2334499	HCCA2	C/T	0.45	T	(Kong et al. 2009)
rs7593730	RBMS1	C/T	0.33	C	(Qi et al. 2010)
rs7578326	KIAA1486	A/G	0.36	A	(Voight et al. 2010)
rs10010131	WFS1	G/A	0.4	G	(Sandhu et al. 2007)
rs864745	JAZF1	A/G	0.499	A	(Zeggini et al. 2008)
rs13266634	SLC30A8	C/T	0.35	C	(Dupuis et al. 2010; Saxena et al. 2007)
rs7754840	CDKAL1	G/C	0.31	C	(Saxena et al. 2007)
rs10440833	CDKAL1	T/A	0.29	A	(Voight et al. 2010)
rs4607103	ADAMTS9	C/T	0.24	C	(Zeggini et al. 2008)
rs6795735	ADAMTS9	C/T	0.41	C	(Voight et al. 2010)
rs12779790	CAMK1D	A/G	0.18	G	(Zeggini et al. 2008)
rs10811661	CDKN2A/B	T/C	0.17	T	(Saxena et al. 2007)
rs1111875	HHEX	G/A	0.47	G	(Saxena et al. 2007)
rs5219	KCNJ11	G/A	0.47	A	(Gloyn et al. 2003)
rs1153188	DCD	T/A	0.27	T	(Zeggini et al. 2008)
rs10830963	MTNR1B	C/G	0.3	G	(Dupuis et al. 2010)
rs1387153	MTNR1B	C/T	0.28	T	(Voight et al. 2010)
rs10923931	NOTCH2	G/T	0.11	T	(Zeggini et al. 2008)

rs7578597	THADA	T/C	0.1	T	(Zeggini et al. 2008)
rs7961581	TSPAN8	T/C	0.27	C	(Zeggini et al. 2008)
rs2237895	KCNQ1	A/C	0.4	C	(Unoki et al. 2008)
rs231362	KCNQ1	G/A	0.48	G	(Voight et al. 2010)
rs11708067	ADCY5	A/G	0.22	A	(Dupuis et al. 2010)
rs4402960	IGF2BP2	A/C	0.3	C	(Saxena et al. 2007)
rs9472138	VEGFA	C/T	0.28	T	(Zeggini et al. 2008)
rs10401969	CILP2	T/C	0.08	C	(Morris et al. 2012)
rs10842994	KLHDC5	C/T	0.2	C	(Morris et al. 2012)
rs12571751	ZMIZ1	A/G	0.48	A	(Morris et al. 2012)
rs12970134	MC4R	G/A	0.27	A	(Morris et al. 2012)
rs13389219	GRB14	C/T	0.4	C	(Morris et al. 2012)
rs459193	ANKRD55	G/A	0.3	G	(Morris et al. 2012)
rs516946	ANK1	C/T	0.24	C	(Morris et al. 2012)
rs7177055	HMG20A	A/G	0.32	A	(Morris et al. 2012)
rs7202877	BCAR1	T/G	0.11	T	(Morris et al. 2012)
rs11063069	CCND2	A/G	0.21	G	(Morris et al. 2012)
rs7501939	HNF1B	C/T	0.39	T	(Gudmundsson et al. 2007)
rs4430796	HNF1B	A/G	0.47	G	(Gudmundsson et al. 2007)
rs757110	ABCC8	T/G	0.29	G	(Hani et al. 1998)
rs8108269	GIPR	T/G	0.31	G	(Morris et al. 2012)

Table S2. Canonical WNT signaling genes

Gene Symbol	Gene Name	Source
APC	adenomatous polyposis coli protein	KEGG pathway - Wnt signaling pathway
APC2	adenomatous polyposis coli protein 2	KEGG pathway - Wnt signaling pathway
AXIN1	axin-1 isoform b,axin-1 isoform a	KEGG pathway - Wnt signaling pathway
AXIN2	axin-2	KEGG pathway - Wnt signaling pathway
CER1	cerberus precursor	KEGG pathway - Wnt signaling pathway
CHD8	chromodomain-helicase-DNA-binding protein 8	KEGG pathway - Wnt signaling pathway
CHP1	calcineurin B homologous protein 1	KEGG pathway - Wnt signaling pathway
CHP2	calcineurin B homologous protein 2	KEGG pathway - Wnt signaling pathway
CREBBP	CREB-binding protein	KEGG pathway - Wnt signaling pathway
CSNK1A1	casein kinase I isoform alpha isoform 1	KEGG pathway - Wnt signaling pathway
CSNK1A1L	casein kinase I isoform alpha-like	KEGG pathway - Wnt signaling pathway
CSNK1E	casein kinase I isoform epsilon	KEGG pathway - Wnt signaling pathway
CSNK2A1	casein kinase II subunit alpha	KEGG pathway - Wnt signaling pathway
CSNK2A2	casein kinase II subunit alpha	KEGG pathway - Wnt signaling pathway
CSNK2B	casein kinase II subunit beta	KEGG pathway - Wnt signaling pathway
CTBP1	C-terminal-binding protein 1	KEGG pathway - Wnt signaling pathway
CTBP2	C-terminal-binding protein 2	KEGG pathway - Wnt signaling pathway
CTNNB1	catenin beta-1	KEGG pathway - Wnt signaling pathway
CTNNBIP1	beta-catenin-interacting protein 1	KEGG pathway - Wnt signaling pathway
CUL1	cullin-1	KEGG pathway - Wnt signaling pathway
CXXC4	CXXC-type zinc finger protein 4	KEGG pathway - Wnt signaling pathway
DKK1	dickkopf-related protein 1 precursor	KEGG pathway - Wnt signaling pathway
DKK2	dickkopf-related protein 2 precursor	KEGG pathway - Wnt signaling pathway

DKK4	dickkopf-related protein 4 precursor	KEGG pathway - Wnt signaling pathway
DVL1	segment polarity protein dishevelled homolog DVL-1	KEGG pathway - Wnt signaling pathway
DVL2	segment polarity protein dishevelled homolog DVL-2	KEGG pathway - Wnt signaling pathway
DVL3	segment polarity protein dishevelled homolog DVL-3	KEGG pathway - Wnt signaling pathway
EP300	histone acetyltransferase p300	KEGG pathway - Wnt signaling pathway
FBXW11	F-box/WD repeat-containing protein 11	KEGG pathway - Wnt signaling pathway
FRAT1	proto-oncogene FRAT1	KEGG pathway - Wnt signaling pathway
FRAT2	GSK-3-binding protein FRAT2	KEGG pathway - Wnt signaling pathway
FZD1	frizzled-1	KEGG pathway - Wnt signaling pathway
FZD10	frizzled-10 precursor	KEGG pathway - Wnt signaling pathway
FZD2	frizzled-2 precursor	KEGG pathway - Wnt signaling pathway
FZD3	frizzled-3 precursor	KEGG pathway - Wnt signaling pathway
FZD4	frizzled-4 precursor	KEGG pathway - Wnt signaling pathway
FZD5	frizzled-5 precursor	KEGG pathway - Wnt signaling pathway
FZD6	frizzled-6	KEGG pathway - Wnt signaling pathway
FZD7	frizzled-7 precursor	KEGG pathway - Wnt signaling pathway
FZD8	frizzled-8 precursor	KEGG pathway - Wnt signaling pathway
FZD9	frizzled-9 precursor	KEGG pathway - Wnt signaling pathway
GSK3B	glycogen synthase kinase-3 beta	KEGG pathway - Wnt signaling pathway
HHEX	hematopoietically expressed homeobox	PubMed ID: 15581885
HNF1A	HNF1 homeobox A	PubMed ID: 16291789, 16325796
LEF1	lymphoid enhancer-binding factor 1	KEGG pathway - Wnt signaling pathway
LRP5	low-density lipoprotein receptor-related protein 5	KEGG pathway - Wnt signaling pathway
LRP6	low-density lipoprotein receptor-related protein 6 precursor	KEGG pathway - Wnt signaling pathway
MAP3K7	mitogen-activated protein kinase kinase kinase 7	KEGG pathway - Wnt signaling pathway

MAPK10	mitogen-activated protein kinase 10	KEGG pathway - Wnt signaling pathway
MAPK8	mitogen-activated protein kinase 8	KEGG pathway - Wnt signaling pathway
MAPK9	mitogen-activated protein kinase 9	KEGG pathway - Wnt signaling pathway
NKD1	protein naked cuticle homolog 1	KEGG pathway - Wnt signaling pathway
NKD2	protein naked cuticle homolog 2	KEGG pathway - Wnt signaling pathway
NLK	serine/threonine-protein kinase NLK	KEGG pathway - Wnt signaling pathway
NOTCH2	neurogenic locus notch homolog protein 2	PubMed ID: 18703315
PORCN	protein-serine O-palmitoleoyltransferase porcupine	KEGG pathway - Wnt signaling pathway
PPARD	peroxisome proliferator-activated receptor delta	KEGG pathway - Wnt signaling pathway
PPARG	peroxisome proliferator activated receptor gamma	PubMed ID: 15308623, 17888405, 19141617, 19307559, 16835228, 15665104
PPP2CA	serine/threonine-protein phosphatase 2A catalytic subunit alpha isoform	KEGG pathway - Wnt signaling pathway
PPP2CB	serine/threonine-protein phosphatase 2A catalytic subunit beta isoform	KEGG pathway - Wnt signaling pathway
PPP2R1A	serine/threonine-protein phosphatase 2A 65 kDa regulatory subunit A alpha	KEGG pathway - Wnt signaling pathway
PPP2R1B	serine/threonine-protein phosphatase 2A 65 kDa regulatory subunit A beta	KEGG pathway - Wnt signaling pathway
PPP2R5A	serine/threonine-protein phosphatase 2A 56 kDa regulatory subunit alpha	KEGG pathway - Wnt signaling pathway
PPP2R5B	serine/threonine-protein phosphatase 2A 56 kDa regulatory subunit beta	KEGG pathway - Wnt signaling pathway
PPP2R5C	serine/threonine-protein phosphatase 2A 56 kDa regulatory subunit gamma	KEGG pathway - Wnt signaling pathway
PPP2R5D	serine/threonine-protein phosphatase 2A 56 kDa regulatory subunit delta	KEGG pathway - Wnt signaling pathway
PPP2R5E	serine/threonine-protein phosphatase 2A 56 kDa regulatory subunit epsilon	KEGG pathway - Wnt signaling pathway
PPP3CA	serine/threonine-protein phosphatase 2B catalytic subunit alpha isoform	KEGG pathway - Wnt signaling pathway

PPP3CB	serine/threonine-protein phosphatase 2B catalytic subunit beta isoform	KEGG pathway - Wnt signaling pathway
PPP3CC	serine/threonine-protein phosphatase 2B catalytic subunit gamma isoform	KEGG pathway - Wnt signaling pathway
PPP3R1	calcineurin subunit B type 1	KEGG pathway - Wnt signaling pathway
PPP3R2	calcineurin subunit B type 2	KEGG pathway - Wnt signaling pathway
PSEN1	presenilin-1	KEGG pathway - Wnt signaling pathway
RBX1	E3 ubiquitin-protein ligase RBX1	KEGG pathway - Wnt signaling pathway
RUVBL1	ruvB-like 1	KEGG pathway - Wnt signaling pathway
SENP2	sentrin-specific protease 2	KEGG pathway - Wnt signaling pathway
SFRP1	secreted frizzled-related protein 1 precursor	KEGG pathway - Wnt signaling pathway
SFRP2	secreted frizzled-related protein 2 precursor	KEGG pathway - Wnt signaling pathway
SFRP4	secreted frizzled-related protein 4 precursor	KEGG pathway - Wnt signaling pathway
SFRP5	secreted frizzled-related protein 5 precursor	KEGG pathway - Wnt signaling pathway
SKP1	S-phase kinase-associated protein 1	KEGG pathway - Wnt signaling pathway
SMAD2	mothers against decapentaplegic homolog 2	KEGG pathway - Wnt signaling pathway
SMAD3	mothers against decapentaplegic homolog 3	KEGG pathway - Wnt signaling pathway
SMAD4	mothers against decapentaplegic homolog 4	KEGG pathway - Wnt signaling pathway
SOX17	transcription factor SOX-17	KEGG pathway - Wnt signaling pathway
TCF7	transcription factor 7	KEGG pathway - Wnt signaling pathway
TCF7L1	transcription factor 7-like 1	KEGG pathway - Wnt signaling pathway
TCF7L2	transcription factor 7-like 2	KEGG pathway - Wnt signaling pathway
TLE4	transducin-like enhancero of split 4	KEGG pathway - Wnt signaling pathway
WIF1	wnt inhibitory factor 1 precursor	KEGG pathway - Wnt signaling pathway
WNT1	proto-oncogene Wnt-1 precursor	KEGG pathway - Wnt signaling pathway
WNT10A	protein Wnt-10a precursor	KEGG pathway - Wnt signaling pathway

WNT10B	protein Wnt-10b precursor	KEGG pathway - Wnt signaling pathway
WNT11	protein Wnt-11 precursor	KEGG pathway - Wnt signaling pathway
WNT16	protein Wnt-16	KEGG pathway - Wnt signaling pathway
WNT2	protein Wnt-2 precursor	KEGG pathway - Wnt signaling pathway
WNT2B	protein Wnt-2b isoform WNT-2B	KEGG pathway - Wnt signaling pathway
WNT3	proto-oncogene Wnt-3 precursor	KEGG pathway - Wnt signaling pathway
WNT3A	protein Wnt-3a precursor	KEGG pathway - Wnt signaling pathway
WNT4	protein Wnt-4 precursor	KEGG pathway - Wnt signaling pathway
WNT5A	protein Wnt-5a	KEGG pathway - Wnt signaling pathway
WNT5B	protein Wnt-5b precursor	KEGG pathway - Wnt signaling pathway
WNT6	protein Wnt-6 precursor	KEGG pathway - Wnt signaling pathway
WNT7A	protein Wnt-7a precursor	KEGG pathway - Wnt signaling pathway
WNT7B	protein Wnt-7b precursor	KEGG pathway - Wnt signaling pathway
WNT8A	protein Wnt-8a	KEGG pathway - Wnt signaling pathway
WNT8B	protein Wnt-8b precursor	KEGG pathway - Wnt signaling pathway
WNT9A	protein Wnt-9a precursor	KEGG pathway - Wnt signaling pathway
WNT9B	protein Wnt-9b precursor	KEGG pathway - Wnt signaling pathway
ZBED3	zinc finger BED domain-containing protein 3	PubMed ID: 19141611

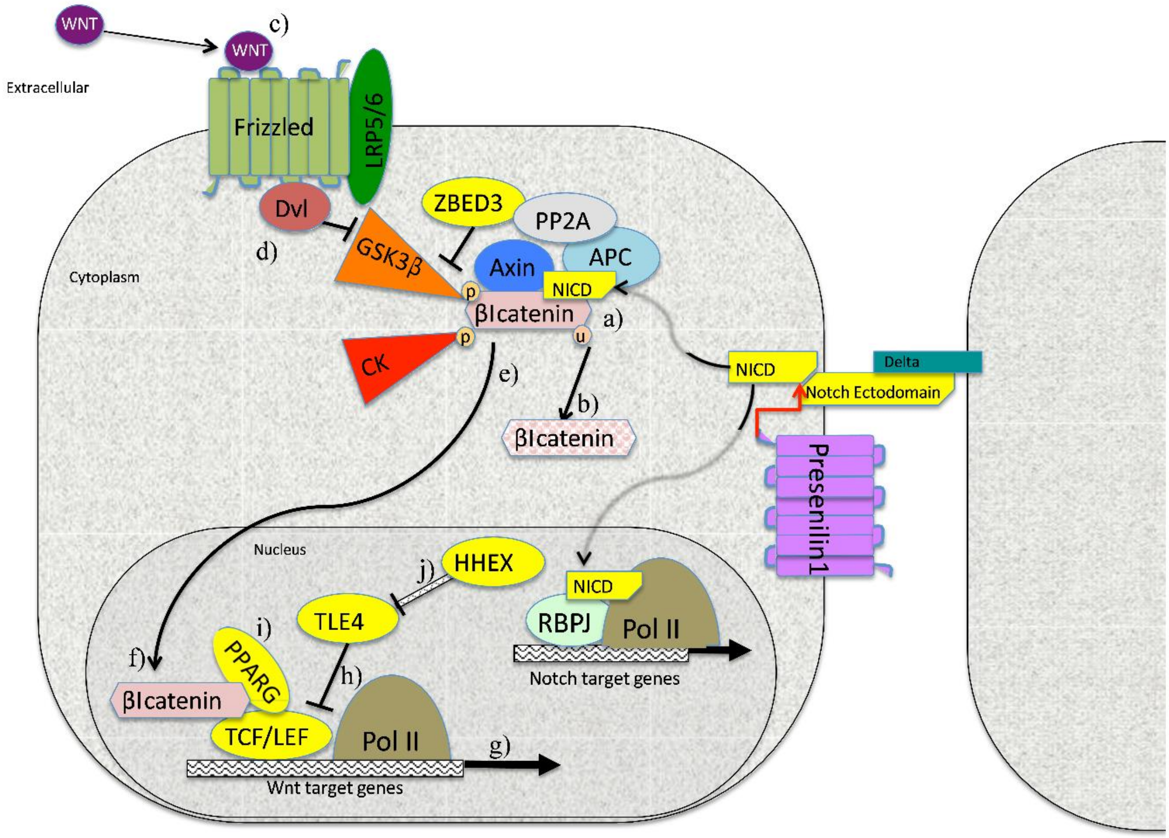

Figure S1. Functional positioning of ZBED3, TLE4, TCF7L2, HNF1A, HHEX, PPARG and NOTCH2 within the WNT Canonical pathway

A total of 9 type 2 diabetes associated genes were annotated to the WNT signaling pathway and of these 7 genes were annotated upstream of TCF7L2 including *HHEX*, *HNF1A*, *NOTCH2* , *TLE4*, *ZBED3* and *PPARG* in addition to *TCF7L2*.When WNT receptor ligands are absent, transcriptional cofactor β-catenin is bound to a destruction complex established around the Axin/APC scaffolding complex (a), (only major components shown: GSK3β, Glycogen synthase kinase 3; CK, Casein kinases; PP2A, Protein phosphatase 2A; APC, Adenomatous polyposis coli). β-catenin is regulated not only through changes to its cytoplasmic concentration, but also through its cellular localization and extensive protein modification. The Axin degradation complex marks β-catenin for proteolysis (b) via phosphorylation and ubiquitination. When ligands bind to the Frizzled-LRP5/6 receptor complex (c), it activates Dishevelled (Dvl) in the cytoplasm of that cell (d). Dvl in turn promotes the attachment of the Axin/APC destruction complex to the Frizzled-LRP5/6 (LRP, Low density lipoprotein receptor-related protein) receptor complex followed by LRP5/6-mediated breakdown of Axin and concomitant release of β-catenin (e) (Logan and Nusse

2004; Tolwinski and Wieschaus 2004). Zinc-finger BED domain-containing 3 (ZBED3) (Chen et al. 2009) binds to Axin leading to inhibition of GSK3β-mediated β-catenin phosphorylation and resulting in cytoplasmic accumulation of free β-catenin, that subsequently translocates to the nucleus where it binds to T-cell factor/lymphoid enhancer factor (TCF/LEF, e.g. TCF7L2 a.k.a. TCF4, HNF1A a.k.a TCF1) (Grumolato et al. 2013) family of transcription modulator complexes on WNT target gene promoters (f) to activate their transcription (g). Factors such as TLE1, TLE2, TLE3 and TLE4 repress the latter transactivation mediated by TCF/LEF complexes and β-catenin (h). Transcription factor PPARG interacts with β-catenin and TCF7L2 (i), but also appears to be a target of the WNT pathway in cancer cells (Jansson et al. 2005; Takada et al. 2009). HHEX, on the other hand wields its enhancing action on WNT signaling by repression of *TLE4* expression (j) (Zamparini et al. 2006). The direct NOTCH-signaling pathway involves interaction of NOTCH with its ligand Delta, then NOTCH undergoes proteolytic cleavage by Presenilin releasing the Notch Intra-Cellular Domain (NICD) (Schroeter et al. 1998), which enters the nucleus and interacts with RBPJ to regulate transcription of specific NOTCH target genes. Convergence between NOTCH signaling and WNT signaling appears to mostly mediate cell fate (Miyamoto et al. 2003; Schroeter et al. 1998). However, there are several pieces of evidence supporting interaction between WNT and NOTCH signaling pathways upstream of gene transcription through which NOTCH downregulates WNT signaling (Hayward et al. 2006; Hayward et al. 2005; Hayward et al. 2008; Katoh and Katoh 2006; Li et al. 2012). β-catenin interacts directly with, and is modulated by, the NOTCH receptor in a ligand independent manner (Hayward et al. 2005; Sanders et al. 2009); there is also evidence for a functional interaction between Axin and ACP in fine-tuning the intracellular traffic of NOTCH (Hayward et al. 2006; Munoz-Descalzo et al. 2011). Dvl also interacts with NOTCH (Carmena et al. 2006) and GSK3 can also phosphorylate NOTCH (Espinosa et al. 2003; Foltz et al. 2002). In addition to proteolytic processing of NOTCH there is evidence that presenilin 1 also associates with β-catenin (Soriano et al. 2001). This supports the notion that WNT and NOTCH signaling are an integrated functional module along with the adherens junctions/cadherin pathway (not shown) regulating β-catenin activity and localization and consequently influencing cell fate of physically adjacent cells.

References

Carmena A, Speicher S, Baylies M (2006) The PDZ protein Canoe/AF-6 links Ras-MAPK, Notch and Wingless/Wnt signaling pathways by directly interacting with Ras, Notch and Dishevelled. PLoS One 1:e66. doi:10.1371/journal.pone.0000066

Chen T et al. (2009) Identification of zinc-finger BED domain-containing 3 (Zbed3) as a novel Axin-interacting protein that activates Wnt/beta-catenin signaling. The Journal of biological chemistry 284:6683-6689. doi:10.1074/jbc.M807753200

Dupuis J et al. (2010) New genetic loci implicated in fasting glucose homeostasis and their impact on type 2 diabetes risk. Nature genetics 42:105-116. doi:10.1038/ng.520

Espinosa L, Ingles-Esteve J, Aguilera C, Bigas A (2003) Phosphorylation by glycogen synthase kinase-3 beta down-regulates Notch activity, a link for Notch and Wnt pathways. The Journal of biological chemistry 278:32227-32235. doi:10.1074/jbc.M304001200

Foltz DR, Santiago MC, Berechid BE, Nye JS (2002) Glycogen synthase kinase-3beta modulates notch signaling and stability. Curr Biol 12:1006-1011

Frayling TM et al. (2007) A common variant in the FTO gene is associated with body mass index and predisposes to childhood and adult obesity. Science 316:889-894. doi:10.1126/science.1141634

Gloyn AL et al. (2003) Large-scale association studies of variants in genes encoding the pancreatic beta-cell KATP channel subunits Kir6.2 (KCNJ11) and SUR1 (ABCC8) confirm that the KCNJ11 E23K variant is associated with type 2 diabetes. Diabetes 52:568-572

Grant SF et al. (2006) Variant of transcription factor 7-like 2 (TCF7L2) gene confers risk of type 2 diabetes. Nature genetics 38:320-323. doi:10.1038/ng1732

Grumolato L et al. (2013) beta-Catenin-independent activation of TCF1/LEF1 in human hematopoietic tumor cells through interaction with ATF2 transcription factors. PLoS genetics 9:e1003603. doi:10.1371/journal.pgen.1003603

Gudmundsson J et al. (2007) Two variants on chromosome 17 confer prostate cancer risk, and the one in TCF2 protects against type 2 diabetes. Nature genetics 39:977-983. doi:10.1038/ng2062

Hani EH, Boutin P, Durand E, Inoue H, Permutt MA, Velho G, Froguel P (1998) Missense mutations in the pancreatic islet beta cell inwardly rectifying K+ channel gene (KIR6.2/BIR): a meta-analysis suggests a role in the polygenic basis of Type II diabetes mellitus in Caucasians. Diabetologia 41:1511-1515. doi:10.1007/s001250051098

Hayward P, Balayo T, Martinez Arias A (2006) Notch synergizes with axin to regulate the activity of armadillo in Drosophila. Developmental dynamics : an official publication of the American Association of Anatomists 235:2656-2666. doi:10.1002/dvdy.20902

Hayward P, Brennan K, Sanders P, Balayo T, DasGupta R, Perrimon N, Martinez Arias A (2005) Notch modulates Wnt signalling by associating with Armadillo/beta-catenin and regulating its transcriptional activity. Development 132:1819-1830. doi:10.1242/dev.01724

Hayward P, Kalmar T, Arias AM (2008) Wnt/Notch signalling and information processing during development. Development 135:411-424. doi:10.1242/dev.000505

Jansson EA, Are A, Greicius G, Kuo IC, Kelly D, Arulampalam V, Pettersson S (2005) The Wnt/beta-catenin signaling pathway targets PPARgamma activity in colon cancer cells. Proc Natl Acad Sci U S A 102:1460-1465. doi:10.1073/pnas.0405928102

Katoh M, Katoh M (2006) NUMB is a break of WNT-Notch signaling cycle. International journal of molecular medicine 18:517-521

Kong A et al. (2009) Parental origin of sequence variants associated with complex diseases. Nature 462:868-874. doi:10.1038/nature08625

Li B et al. (2012) Interaction of Wnt/beta-catenin and notch signaling in the early stage of cardiac differentiation of P19CL6 cells. Journal of cellular biochemistry 113:629-639. doi:10.1002/jcb.23390

Logan CY, Nusse R (2004) The Wnt signaling pathway in development and disease. Annual review of cell and developmental biology 20:781-810. doi:10.1146/annurev.cellbio.20.010403.113126

Miyamoto Y et al. (2003) Notch mediates TGF alpha-induced changes in epithelial differentiation during pancreatic tumorigenesis. Cancer cell 3:565-576

Morris AP et al. (2012) Large-scale association analysis provides insights into the genetic architecture and pathophysiology of type 2 diabetes. Nature genetics 44:981-990. doi:10.1038/ng.2383
Munoz-Descalzo S, Tkocz K, Balayo T, Arias AM (2011) Modulation of the ligand-independent traffic of Notch by Axin and Apc contributes to the activation of Armadillo in Drosophila. Development 138:1501-1506. doi:10.1242/dev.061309
Qi L et al. (2010) Genetic variants at 2q24 are associated with susceptibility to type 2 diabetes. Human molecular genetics 19:2706-2715. doi:10.1093/hmg/ddq156
Rung J et al. (2009) Genetic variant near IRS1 is associated with type 2 diabetes, insulin resistance and hyperinsulinemia. Nature genetics 41:1110-1115. doi:10.1038/ng.443
Sanders PG, Munoz-Descalzo S, Balayo T, Wirtz-Peitz F, Hayward P, Arias AM (2009) Ligand-independent traffic of Notch buffers activated Armadillo in Drosophila. PLoS biology 7:e1000169. doi:10.1371/journal.pbio.1000169
Sandhu MS et al. (2007) Common variants in WFS1 confer risk of type 2 diabetes. Nature genetics 39:951-953. doi:10.1038/ng2067
Saxena R et al. (2007) Genome-wide association analysis identifies loci for type 2 diabetes and triglyceride levels. Science 316:1331-1336. doi:10.1126/science.1142358
Schroeter EH, Kisslinger JA, Kopan R (1998) Notch-1 signalling requires ligand-induced proteolytic release of intracellular domain. Nature 393:382-386. doi:10.1038/30756
Soriano S, Kang DE, Fu M, Pestell R, Chevallier N, Zheng H, Koo EH (2001) Presenilin 1 negatively regulates beta-catenin/T cell factor/lymphoid enhancer factor-1 signaling independently of beta-amyloid precursor protein and notch processing. The Journal of cell biology 152:785-794
Takada I, Kouzmenko AP, Kato S (2009) Wnt and PPARgamma signaling in osteoblastogenesis and adipogenesis. Nature reviews Rheumatology 5:442-447. doi:10.1038/nrrheum.2009.137
Tolwinski NS, Wieschaus E (2004) Rethinking WNT signaling. Trends in genetics : TIG 20:177-181. doi:10.1016/j.tig.2004.02.003
Unoki H et al. (2008) SNPs in KCNQ1 are associated with susceptibility to type 2 diabetes in East Asian and European populations. Nature genetics 40:1098-1102. doi:10.1038/ng.208
Voight BF et al. (2010) Twelve type 2 diabetes susceptibility loci identified through large-scale association analysis. Nature genetics 42:579-589. doi:10.1038/ng.609
Zamparini AL, Watts T, Gardner CE, Tomlinson SR, Johnston GI, Brickman JM (2006) Hex acts with beta-catenin to regulate anteroposterior patterning via a Groucho-related co-repressor and Nodal. Development 133:3709-3722. doi:10.1242/dev.02516
Zeggini E et al. (2008) Meta-analysis of genome-wide association data and large-scale replication identifies additional susceptibility loci for type 2 diabetes. Nature genetics 40:638-645. doi:10.1038/ng.120
